# Evaluating User Interactions and Adoption Patterns of Generative AI in Health Care Occupations Using Claude: Cross-Sectional Study

**DOI:** 10.2196/73918

**Published:** 2025-05-30

**Authors:** Gabriel Alain, James Crick, Ella Snead, Catherine C Quatman-Yates, Carmen E Quatman

**Affiliations:** 1 School of Health and Rehabilitation Sciences, College of Medicine, The Ohio State University, Columbus, OH, 43210, United States, 1 614-292-1706; 2 The Center for the Advancement of Team Science, Analytics, and Systems Thinking in Health Services and Implementation Science Research (CATALYST), College of Medicine, The Ohio State University, Columbus, OH, United States; 3 Division of Physical Therapy, College of Medicine, The Ohio State University, Columbus, OH, United States; 4 The Ohio State University Sports Medicine Research Institute, Columbus, OH, United States; 5 Department of Emergency Medicine, College of Medicine, The Ohio State University, Columbus, OH, United States; 6 Department of Orthopaedics, Division of Trauma, The Ohio State University, Columbus, OH, United States

**Keywords:** communication, humans, artificial intelligence, language, machine learning, self-management, information-seeking behavior, outcome assessment/health care, referral and consultation, patient care, workflow, patient participation, health literacy

## Abstract

**Background:**

Generative artificial intelligence (GenAI) systems like Anthropic’s Claude and OpenAI’s ChatGPT are rapidly being adopted in various sectors, including health care, offering potential benefits for clinical support, administrative efficiency, and patient information access. However, real-world adoption patterns and the extent to which GenAI is used for health care–related tasks remain poorly understood and distinct from performance benchmarks in controlled settings. Understanding these organic usage patterns is key for assessing GenAI’s impact on health care delivery and patient-provider dynamics.

**Objective:**

This study aimed to quantify the real-world frequency and scope of health care–related tasks performed using Anthropic’s Claude GenAI. We sought to (1) measure the proportion of Claude interactions related to health care tasks versus other domains; (2) identify specific health care occupations (as per O*NET classifications) with high associated interaction volumes; (3) assess the breadth of task adoption within roles using a “digital adoption rate”; and (4) interpret these findings considering the inherent ambiguity regarding user identity (ie, professionals vs public) in the dataset.

**Methods:**

We performed a cross-sectional analysis of more than 4 million anonymized user conversations with Claude (ie, including both free and pro subscribers) from December 2024 to January 2025, using a publicly available dataset from Anthropic’s Economic Index research. Interactions were preclassified by Anthropic’s proprietary Clio model into standardized occupational tasks mapped to the US Department of Labor’s O*NET database. The dataset did not allow differentiation between health care professionals and the general public as users. We focused on interactions mapped to O*NET Healthcare Practitioners and Technical Occupations. Main outcomes included the proportion of interactions per health care occupation, proportion of overall health care interaction versus other categories, and the digital adoption rate (ie, distinct tasks performed via GenAI divided by the total possible tasks per occupation).

**Results:**

Health care–related tasks accounted for 2.58% of total analyzed GenAI conversations, significantly lower than domains such as computing (37.22%). Within health care, interaction frequency varied notably by role. Occupations emphasizing patient education and guidance exhibited the highest proportion, including dietitians and nutritionists (6.61% of health care conversations), nurse practitioners (5.63%), music therapists (4.54%), and clinical nurse specialists (4.53%). Digital adoption rates (task breadth) ranged widely across top health care roles (13.33%‐65%), averaging 16.92%, below the global average (21.13%). Tasks associated with medical records and health information technicians had the highest adoption rate (65.0%).

**Conclusions:**

GenAI tools are being adopted for a measurable subset of health care–related tasks, with usage concentrated in specific, often patient-facing roles. The critical limitation of user anonymity prevents definitive conclusions regarding whether usage primarily reflects patient information–seeking behavior (potentially driven by access needs) or professional workflow assistance. This ambiguity necessitates caution when interpreting current GenAI adoption. Our findings emphasize the urgent need for strategies addressing potential impacts on clinical workflows, patient decision-making, information quality, and health equity. Future research must aim to differentiate user types, while stakeholders should develop targeted guidance for both safe patient use and responsible professional integration.

## Introduction

Generative artificial intelligence (GenAI) systems, developed by organizations such as Anthropic (Claude), OpenAI (ChatGPT), Google (Gemini), Microsoft (Copilot), and Meta (Llama), represent a significant technological shift and are rapidly being adopted in diverse sectors globally at an unprecedented speed [1]. Health care represents a domain where GenAI holds considerable transformative potential. Proposed applications range from enhancing clinical decision-making support [2,3] and potentially automating high-skilled cognitive tasks distinct from previous software capabilities [4], to streamlining burdensome administrative workflows and empowering patients with more accessible, tailored health information [2,3]. The rapid proliferation and increasing sophistication of these tools necessitate a clear understanding of their current role and impact within the health care ecosystem.

Initial research has started to explore the capabilities of GenAI in health care contexts, often demonstrating performance on par with, or even exceeding that of human clinicians on specific, well-defined tasks such as diagnostic reasoning or medical knowledge recall [5-7]. For instance, studies have compared GenAI outputs to physician performance in complex diagnostic challenges [6] and clinical reasoning assessments [5]. Furthermore, user trust plays a critical role in the adoption of these AI tools [8], adding another layer to understanding real-world uptake. However, these studies typically occur in controlled or experimental settings, focusing on benchmark performance rather than quantifying the extent and nature of GenAI’s spontaneous, real-world application across the breadth of health care activities.

Consequently, a significant gap exists between understanding GenAI’s potential capabilities and characterizing its actual adoption patterns and usage frequency for health care–related tasks by both professionals and the public. Studies have confirmed that rapid AI adoption is occurring across the economy [1,9], highlighting the urgency of understanding its domain-specific implications. This widespread availability and adoption of GenAI tools raise many questions about their real-world integration [10]. As individuals increasingly turn to free and widely available GenAI models, how frequently are these interactions related to health care? Are certain clinical roles or types of health care tasks experiencing disproportionately high engagement via GenAI? Given these questions, we sought to move beyond capability assessment and provide empirical data on the current landscape of GenAI usage for tasks associated with health care occupations.

Using Anthropic’s foundational “Economic Index” research which provided a broad overview of artificial intelligence (AI) usage across various economic sectors [11], this cross-sectional study aimed to conduct a more focused analysis specifically within the health care domain. Leveraging the same large-scale, anonymized dataset of user interactions with Claude, our primary objectives were to (1) quantify the overall proportion of GenAI interactions dedicated to tasks associated with health care occupations, as defined by the US Department of Labor’s [12] O*NET database [13]; (2) identify which specific health care roles exhibit the highest frequency of associated task interactions; (3) introduce and assess a novel metric—the “digital adoption rate,”—representing the breadth of GenAI adoption within these roles based on the variety of distinct occupational tasks performed using the AI; and (4) contextualize health care–related usage by comparing it with usage patterns across other major occupational sectors.

## Methods

### Data Source and Study Design

We leveraged a publicly available, privacy-preserving aggregated dataset derived from over 4 million anonymized conversations with Claude from free and pro subscribers, released as part of Anthropic’s “Economic Index” research [11]. This dataset reflects a broad spectrum of real-world user interactions, offering an ecologically valid basis for examining GenAI engagement with professional tasks.

### Occupational Task Mapping

**Figure 1.**
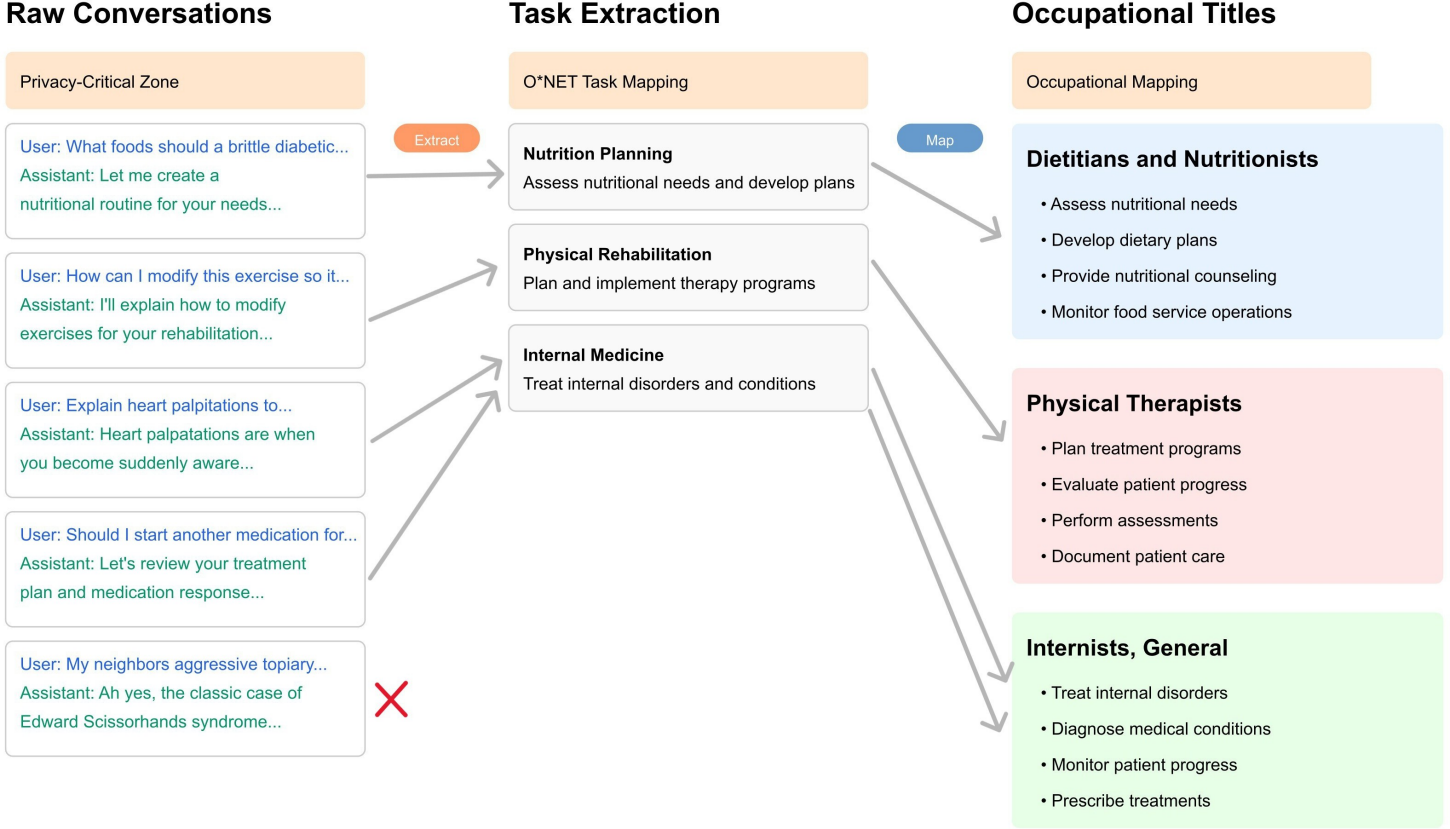
Framework for mapping GenAI conversations to O*NET tasks and occupational categories.

Our analysis used the premapped dataset created and open-sourced by Anthropic, which contains conversations already mapped to standardized occupational tasks as defined by the US Department of Labor’s O*NET database. Anthropic’s team used their proprietary Clio model to create these mappings and validated them through manual review [14]. Specifically, they used hand validation across 150 examples for task hierarchy classifications, finding that 86% of conversations were judged as correctly assigned at the base O*NET task level, 91.3% at the middle level, and 95.3% at the top level of their hierarchical framework [11]. We focused our analysis specifically on the subset of this dataset related to tasks and occupations within the health care domain. Figure 1 illustrates the Clio mapping methodology that underpins the dataset in this study.

### Metrics and Outcomes

- Occupation-specific GenAI usage: We computed the proportion of total GenAI conversations attributable to each O*NET Healthcare Practitioners and Technical Occupation.
- Global occupational categories: We compared health care’s share of GenAI usage against other occupational categories (eg, computing, arts and media, education) to contextualize overall representation.
- Digital adoption rate: We calculated the ratio of distinct tasks performed with GenAI to total distinct potential tasks for each occupation. For instance, if an occupation had 20 potential tasks in the O*NET database and 5 of them appeared in GenAI conversations, the adoption rate was 25%. This analysis was conducted for the top 25 health care practitioners and technical occupations.

### Statistical Analysis

Descriptive statistics summarized GenAI usage proportions and adoption rates. We used simple comparisons (eg, ratio of usage in health care vs other categories) to maximize interpretability while highlighting relative differences. All analyses were performed using R software (version 4.3.1; R Foundation for Statistical Computing) [15].

### Ethical Considerations

Ethics Committee of The Ohio State University determined that our study did not qualify as human subjects research requiring review as no identifiable personal data were included in the dataset. All conversations were preanonymized and aggregated, and the data were made public by Anthropic on February 10, 2025 [16].

## Results

### Overall Health care Usage

Tasks mapped to Health care Practitioners and Technical Occupations collectively represented 2.58% of all GenAI conversations (Table 1 and Multimedia Appendix 1). In contrast, computing‐related fields accounted for 37.22%, followed by arts and media (10.28%) and education (9.33%).

**Table 1.** Distribution of GenAI conversations based on O*NET major occupational category. More than 4 million anonymized Claude conversations (December 2024 – January 2025) were mapped to each top-level occupational group.

Occupational categories	Proportion of anonymized Claude conversations (global %)^a^
Computer and mathematics	37.22
Arts, design, entertainment, sports, and media	10.28
Educational instruction and library	9.33
Office and administrative support	7.87
Life, physical, and social science	6.38
Business and financial operations	5.93
Architecture and engineering	4.51
Management	4.48
Production	2.93
Health care practitioners and technical	2.58
Sales and related	2.27
Community and social service	2.07
Legal	0.90
Installation, maintenance, and repair	0.72
Food preparation and serving-related	0.53
Personal care and service	0.46
Construction and extraction	0.42
Protective service	0.38
Transportation and material moving	0.28
Health care support	0.27
Building and grounds cleaning and maintenance	0.10
Farming, fishing, and forestry	0.07

a Absolute values (n) are not available due to privacy-preserving preprocessing of the source data that involved filtering and aggregation steps which preclude determination of exact conversation counts.

### Within-Health Care Breakdown

In the top 25 list of Health care Practitioners and Technical Occupations by GenAI conversation share, dietitians and nutritionists (6.61%), nurse practitioners (5.63%), music therapists (4.54%), and clinical nurse specialists (4.53%) had the highest usage (Table 2 and Multimedia Appendix 2). Meanwhile, other occupations such as pediatricians (1.38%) and internists (1.29%) showed more modest representation.

**Table 2.** Top 25 health care occupations ranked by proportion of Claude conversations. Health care practitioners and technical occupations with the highest proportion of health care–related GenAI interactions are shown.

Top 25 health care practitioners and technical occupations^a^	Health care–mapped conversations, % (total conversations, %)^b^^c^
Dietitians and nutritionists	6.61 (0.17)
Nurse practitioners	5.63 (0.15)
Music therapists	4.54 (0.12)
Clinical nurse specialists	4.53 (0.12)
Radiologists	4.16 (0.11)
Exercise physiologists	3.91 (0.10)
Respiratory therapists	3.86 (0.10)
Medical records and health information technicians	3.74 (0.10)
Critical care nurses	3.03 (0.08)
Medical and clinical laboratory technicians	2.98 (0.08)
Genetic counselors	2.83 (0.07)
Pharmacists	2.75 (0.07)
Nuclear medicine physicians	2.54 (0.07)
Pathologists	2.52 (0.07)
Advanced practice psychiatric nurses	2.43 (0.06)
Art therapists	2.42 (0.06)
Allergists and immunologists	2.25 (0.06)
Dietetic technicians	2.08 (0.05)
Nurse midwives	1.89 (0.05)
Medical and clinical laboratory technologists	1.88 (0.05)
Physical therapists	1.75 (0.05)
Naturopathic physicians	1.69 (0.04)
Acute care nurses	1.55 (0.04)
General pediatricians	1.38 (0.04)
General internists	1.29 (0.03)

a Health care practitioners and technical occupations with the highest proportion of health care–related GenAI interactions.

b Absolute numbers (n) are not available due to privacy-preserving preprocessing of the source data that involved filtering and aggregation steps which preclude determination of exact conversation counts.

c Values show the proportion of each occupation among health care–mapped conversations and corresponding percentages of total conversations across all domains.

### Adoption Rates

Digital adoption rates for top health care occupations ranged from 13.33% to 65%, with an average of 16.92%, compared to a global task adoption rate of 21.13% (Table 3 and Multimedia Appendix 3). Medical records and health information technicians (65%), nurse midwives (50%), and pediatricians (43.75%) had the highest task adoption rate.

**Table 3.** Digital adoption rates for leading health care occupations. Breadth of GenAI use within the top 25 health care occupations, expressed as the percentage of distinct O*NET tasks observed in Claude conversations were divided by the total distinct tasks defined for that occupation (affected tasks/total tasks).

Top 25 health care practitioners and technical occupation adoption rates	Adoption rate, %, (affected tasks/total tasks)
Medical records and health information technicians	65.00 (13/20)
Nurse midwives	50.00 (10/20)
General pediatricians	43.75 (7/16)
Nurse practitioners	37.04 (10/27)
Dietitians and nutritionists	34.78 (8/23)
Dietetic technicians	33.33 (4/12)
Exercise physiologists	32.00 (8/25)
General internists	31.58 (6/19)
Genetic counselors	31.58 (6/19)
Clinical nurse specialists	31.03 (9/29)
Pharmacists	30.00 (6/20)
Radiologists	27.59 (8/29)
Music therapists	26.67 (8/30)
Pathologists	26.32 (5/19)
Acute care nurses	25.93 (7/27)
Physical therapists	25.00 (6/24)
Naturopathic physicians	25.00 (5/20)
Medical and clinical laboratory technologists	25.00 (4/16)
Allergists and immunologists	25.00 (4/16)
Advanced practice psychiatric nurses	25.00 (6/24)
Art therapists	24.00 (6/25)
Critical care nurses	17.24 (5/29)
Nuclear medicine physicians	15.38 (4/26)
Respiratory therapists	13.64 (3/22)
Medical and clinical laboratory technicians	13.33 (2/15)

## Discussion

### Interpreting GenAI Usage Patterns in Health care Tasks Through Multiple Perspectives

Our analysis reveals significant GenAI interaction volume related to health care tasks, yet concentrated within specific roles, particularly those involving substantial patient interaction, education, and guidance (eg, dietitians, nurse practitioners, therapists). These findings point toward an emerging, complex integration of GenAI into the health care ecosystem. However, the anonymized nature of the data necessitates consideration of multiple interpretations about the users driving this usage and their underlying reasons.

#### Interpretation 1: Patient-Driven Information Seeking and Self-Management

One plausible interpretation is that the observed patterns reflect significant usage by the public. Patients may be turning to GenAI such as Claude, for accessible health information, clarification of medical terminology, lifestyle advice, or even emotional support related to health concerns—tasks commonly associated with roles such as dietitians or nurse practitioners. Such information-seeking behavior aligns with pre-existing trends of patients consulting “Dr. Google” [17] and reflects a potential desire for readily available information [18]. Observed patterns among ChatGPT users seeking health information, who tend to be younger, report poorer health status, and more frequently use transient care settings [19], may further point to this behavior, particularly when timely access to health care professionals is limited [20]. If patients are indeed primary users, this trend could signify a shift toward greater patient engagement and potential empowerment through readily accessible, personalized information. However, this interpretation also carries significant risks: the potential for consuming misinformation or AI “hallucinations,” leading to misguided self-management decisions, delayed consultation with qualified professionals, or increased health anxiety [18]. These risks have been highlighted in studies examining user trust and reliance on chatbots for health advice [8]. The accessibility that drives usage could simultaneously introduce harm if not accompanied by subject matter expertise, digital health literacy, and critical appraisal skills.

#### Interpretation 2: Professional Workflow Assistance and Efficiency

Alternatively, the concentration of usage in these roles could indicate adoption by health care professionals themselves. Dietitians, nurse practitioners, clinical nurse specialists, and others might be leveraging GenAI to enhance their workflow–drafting patient education materials, summarizing complex information, exploring differential diagnoses, generating notes, or staying abreast of new research relevant to patient counseling. These uses suggest potential automation of specific cognitive tasks previously resistant to software solutions [4]. From this perspective, GenAI acts as an efficiency tool, potentially helping overloaded professionals manage demanding workloads within a strained health care system. Indeed, workers in exposed occupations often perceive substantial productivity potential in GenAI tools like ChatGPT [9]. This interpretation suggests a pathway towards augmenting professional capabilities, potentially improving the consistency or breadth of patient communication. Yet, risks exist here as well, including over-reliance on AI outputs that potentially contain inaccuracies or biases, deskilling in core communication or critical thinking tasks through cognitive offloading, and ethical implications of using AI to generate content in patient care without adequate oversight or personalization.

### The Underlying Influence of Health care System Pressures

Regardless of whether patients or professionals are the primary users, the observed patterns may also indicate broader systemic issues within health care. When professional support is not easily or quickly accessible, individuals—both patients and potentially time-pressed professionals—are likely to turn to readily available tools such as GenAI. Findings that users seeking health information from ChatGPT tend to report poorer health and greater use of transient care settings lend credence to this possibility [19]. This suggests that GenAI adoption in health care contexts might be partially driven by unmet needs and access gaps within the current system. Furthermore, even when productivity benefits are perceived, adoption may be hindered by factors such as employer restrictions or the need for training [9]. This “compensatory” use stresses the importance for health care systems to adapt, not only by integrating AI safely, but by addressing the root causes that drive patients or professionals toward potentially unverified AI tools out of necessity.

### Implications for the Patient-Provider Dynamic and Information Hierarchy

The convergence of these possibilities, patients potentially arriving at appointments armed with AI-generated information, and professionals using GenAI to prepare for those encounters, points toward a fundamental reshaping of information hierarchies and the patient-provider relationship. The dynamic shifts from a traditionally asymmetric flow of information to one where both parties may engage with AI-derived knowledge. This shift demands new communication strategies, enhanced critical appraisal skills from both sides, and a shared understanding of GenAI’s current capabilities and limitations [7]. This is particularly relevant given that a significant portion of patients report acting on AI-generated health information, such as changing medications or seeking referrals, and often subsequently discussing the AI output with their physicians [19]. Addressing the potential for GenAI to exacerbate existing health inequities, especially those based on digital access or literacy, is also paramount.

### Future Directions

Health care organizations and regulatory bodies should develop comprehensive frameworks for responsible AI integration. These should include creating evidence-based guidelines for clinical practice integration and establishing rigorous standards for the accuracy of AI-generated health information. Ethical considerations surrounding data privacy, training bias, and the potential for exacerbating health inequities must also be central to any framework for responsible GenAI integration. Future research should investigate whether using GenAI translates into improved health outcomes and how shifts in digital literacy affect patient engagement. Importantly, developing methodologies to better differentiate between professional and lay user interactions with health-related GenAI queries will be essential for accurately interpreting adoption trends and tailoring interventions at scale.

### Limitations

First, we relied on anonymized, aggregated conversations; thus information on user credentials and demographics is not available. Second, the mapping from conversation text to occupational tasks may be imperfect, particularly when laypersons seek professional-grade information. Third, our cross-sectional design provides a single snapshot in time rather than longitudinal trends. Fourth, data from conversations were limited to Claude since Anthropic is the only company that has open-sourced data for analysis. The demographics of Claude users, along with the reasons why users engage with Claude, may or may not be representative across other GenAI tools which could fundamentally bias usage patterns. Finally, we do not know what users did with the information they received from GenAI or how it impacted health outcomes. Future analyses should account for ongoing changes in GenAI capabilities and usage patterns.

### Conclusion

Generative AI is making tangible inroads into health care–related tasks, yet its adoption appears selective, with high interaction volumes associated with patient-facing roles such as dietitians and nurse practitioners. Our analysis, based on interactions with Claude, highlights this emerging pattern but calls attention to a critical ambiguity: whether this usage is primarily driven by patients seeking information and navigating access challenges, by professionals seeking workflow efficiencies, or by a combination of both.

Regardless of the primary user, this integration demands immediate attention. The observed patterns necessitate a shift beyond simply acknowledging GenAI’s potential, toward implementing proactive strategies. Future research must focus on identifying who uses these tools for health care–related tasks and understanding their motivations, potentially through methods that more accurately infer user intent or role. Concurrently, stakeholders including clinicians, educators, developers, and policymakers must develop distinct guidance—resources promoting digital health literacy and critical appraisal for patients, and evidence-based best practices for safe and effective integration into professional clinical workflows. Addressing how systemic health care pressures may be influencing this adoption is also crucial. Ensuring GenAI enhances, rather than compromises, health care delivery requires targeted action now to shape its responsible use before usage patterns solidify.

## Supplementary material

10.2196/73918 Multimedia Appendix 1 Global percentage of GenAI conversations by occupational categories.

10.2196/73918 Multimedia Appendix 2 Top 25 health care practitioners and technical occupational titles by GenAI conversation share. Values in parentheses show each occupation's share of total global GenAI conversations, while the primary percentage represents the share within the health care practitioner group.

10.2196/73918 Multimedia Appendix 3 GenAI task adoption rate for top 25 health care practitioners and technical occupations. Values show each occupation's adoption rate as a percentage of possible tasks (total adopted tasks/total available tasks), with the raw task counts shown in parentheses.

## Abbreviations

AI: artificial intelligence
GenAI: Generative Artificial Intelligence
