# Supplementary figures and images for: Evaluating User Interactions and Adoption Patterns of Generative AI in Health Care Occupations Using Claude: Cross-Sectional Study
Source: J Med Internet Res. 2025 May 30;27:e73918. doi: 10.2196/73918 (PMC12143583; doi:10.2196/73918)

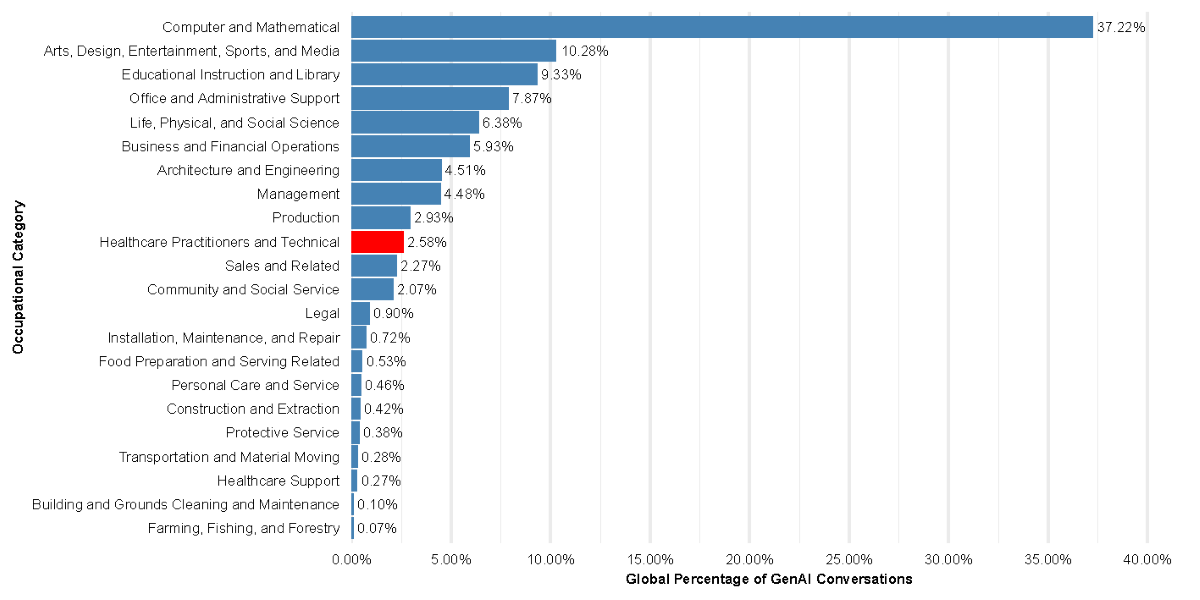

Supplement: Multimedia Appendix 1 [file jmir-v27-e73918-s001.png]

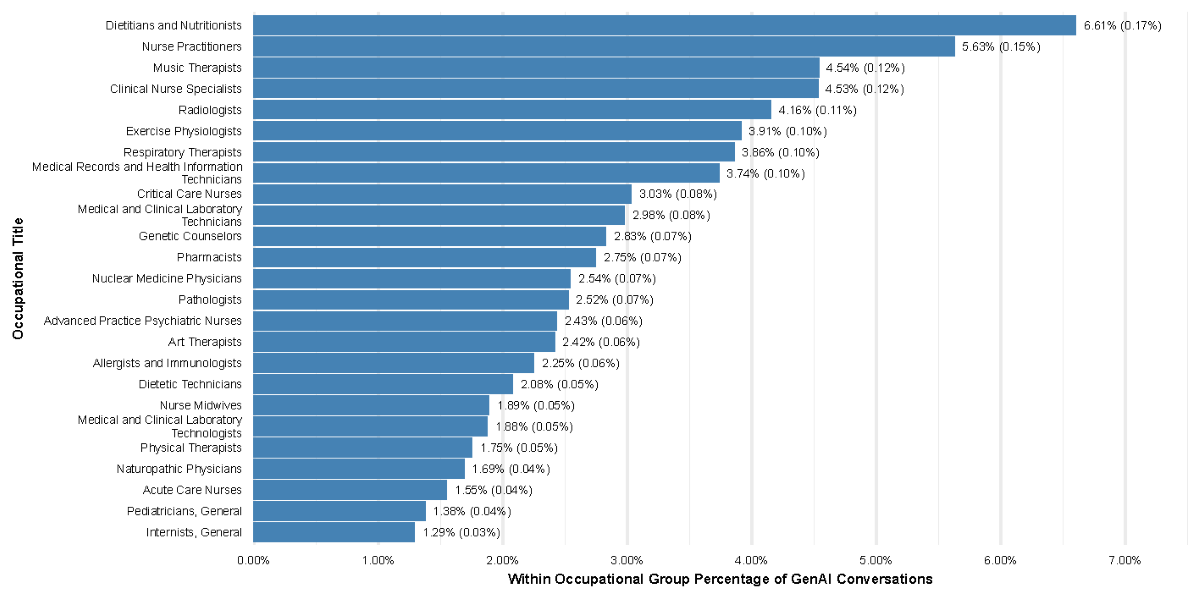

Supplement: Multimedia Appendix 2 [file jmir-v27-e73918-s002.png]

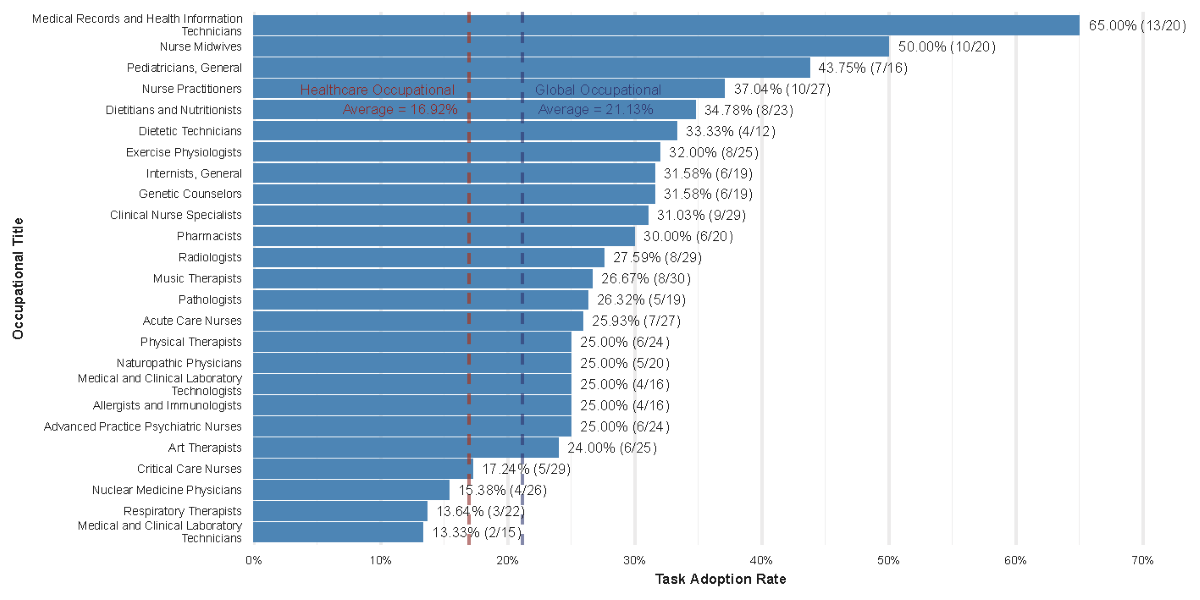

Supplement: Multimedia Appendix 3 [file jmir-v27-e73918-s003.png]
